# Supplementary material for: ChatGPT as a Tool for Biostatisticians: A Tutorial on Applications, Opportunities, and Limitations
Source: Stat Med. 2025 Oct 23;44(23-24):e70263. doi: 10.1002/sim.70263 (PMC12548020; doi:10.1002/sim.70263)
Supplement: Supplementary file 1 — Data S1. Supporting Information. [file SIM-44-0-s001.zip › Supplementary Materials Additional.zip!/3.6_Simulation_Study/3.6_Simulation_Chat8.pdf]

Coverage prob8 - Simulation CI Coverage

Take the role of an expert biostatistician for this chat. The goal is to perform a simulation study to assess the coverage probability of a confidence interval with nominal coverage 95%. The setting is a case control study design, which is a retrospective observational study that compares individuals with a disease (cases) to those without it (controls) to identify potential risk factors or exposures associated with the disease.

We aim at the coverage probability of an appropriate effect size estimate in a parametric model. Propose a suitable exact confidence interval and investigate its empirical coverage under the null hypothesis of no effect. Use 5000 Monte Carlo runs and the sample sizes must vary between 20 and 40. Provide results for ten different settings with different sample sizes and marginal probabilities. Be consize in your answer and do not provide unnecessary descriptions.

```python
import numpy as np
import pandas as pd
from statsmodels.api import Logit, add_constant
from scipy.special import expit
from scipy.stats import norm
import ace_tools as tools

# Simulation parameters
n_simulations = 5000
sample_sizes = [(n_cases, n_controls) for n_cases in range(10, 25, 2) for n_controls in
range(10, 25, 2)][:10]
true_beta = 0.0  # Null hypothesis: no effect
alpha = 0.05

results = []

for n_cases, n_controls in sample_sizes:
    coverage_count = 0

    for _ in range(n_simulations):
        # Generate exposure for controls and cases under null (no association)
        p_exposed_controls = np.random.uniform(0.1, 0.9)
        exposure_controls = np.random.binomial(1, p_exposed_controls, size=n_controls)
        exposure_cases = np.random.binomial(1, p_exposed_controls, size=n_cases)

        # Stack data
        y = np.concatenate([np.ones(n_cases), np.zeros(n_controls)])  # 1 = case, 0 = control
        x = np.concatenate([exposure_cases, exposure_controls])
        X = add_constant(x)

        try:
            model = Logit(y, X).fit(disp=0)
            beta_hat = model.params[1]
            se = model.bse[1]

            ci_lower = beta_hat - norm.ppf(1 - alpha/2) * se
            ci_upper = beta_hat + norm.ppf(1 - alpha/2) * se

            if ci_lower <= true_beta <= ci_upper:
                coverage_count += 1
        except:
            continue

    coverage_probability = coverage_count / n_simulations
```

```
    results.append({
        "n_cases": n_cases,
        "n_controls": n_controls,
        "coverage_probability": coverage_probability
    })

results_df = pd.DataFrame(results)
tools.display_dataframe_to_user(name="Coverage Probability Results", dataframe=results_df)
```

STDOUT/STDERR

```
rn("Maximum Likelihood optimization failed to "
/home/sandbox/.local/lib/python3.11/site-packages/statsmodels/base/model.py:604: ConvergenceWarning: Maximum
  warnings.warn("Maximum Likelihood optimization failed to "
/home/sandbox/.local/lib/python3.11/site-packages/statsmodels/base/model.py:604: ConvergenceWarning: Maximum
  warnings.warn("Maximum Likelihood optimization failed to "
/home/sandbox/.local/lib/python3.11/site-packages/statsmodels/base/model.py:604: ConvergenceWarning: Maximum
  warnings.warn("Maximum Likelihood optimization failed to "
/home/sandbox/.local/lib/python3.11/site-packages/statsmodels/base/model.py:604: ConvergenceWarning: Maximum
  warnings.warn("Maximum Likelihood optimization failed to "
/home/sandbox/.local/lib/python3.11/site-packages/statsmodels/base/model.py:604: ConvergenceWarning: Maximum
  warnings.warn("Maximum Likelihood optimization failed to "
/home/sandbox/.local/lib/python3.11/site-packages/statsmodels/base/model.py:604: ConvergenceWarning: Maximum
  warnings.warn("Maximum Likelihood optimization failed to "
/home/sandbox/.local/lib/python3.11/site-packages/statsmodels/base/model.py:604: ConvergenceWarning: Maximum
  warnings.warn("Maximum Likelihood optimization failed to "
/home/sandbox/.local/lib/python3.11/site-packages/statsmodels/base/model.py:604: ConvergenceWarning: Maximum
  warnings.warn("Maximum Likelihood optimization failed to "
/home/sandbox/.local/lib/python3.11/site-packages/statsmodels/base/model.py:604: ConvergenceWarning: Maximum
  warnings.warn("Maximum Likelihood optimization failed to "
/home/sandbox/.local/lib/python3.11/site-packages/statsmodels/base/model.py:604: ConvergenceWarning: Maximum
  warnings.warn("Maximum Likelihood optimization failed to "
/home/sandbox/.local/lib/python3.11/site-packages/statsmodels/base/model.py:604: ConvergenceWarning: Maximum
  warnings.warn("Maximum Likelihood optimization failed to "
/home/sandbox/.local/lib/python3.11/site-packages/statsmodels/base/model.py:604: ConvergenceWarning: Maximum
  warnings.warn("Maximum Likelihood optimization failed to "
/home/sandbox/.local/lib/python3.11/site-packages/statsmodels/base/model.py:604: ConvergenceWarning: Maximum
  warnings.warn("Maximum Likelihood optimization failed to "
/home/sandbox/.local/lib/python3.11/site-packages/statsmodels/base/model.py:604: ConvergenceWarning: Maximum
  warnings.warn("Maximum Likelihood optimization failed to "
/home/sandbox/.local/lib/python3.11/site-packages/statsmodels/base/model.py:604: ConvergenceWarning: Maximum
  warnings.warn("Maximum Likelihood optimization failed to "
/home/sandbox/.local/lib/python3.11/site-packages/statsmodels/base/model.py:604: ConvergenceWarning: Maximum
  warnings.warn("Maximum Likelihood optimization failed to "
/home/sandbox/.local/lib/python3.11/site-packages/statsmodels/base/model.py:604: ConvergenceWarning: Maximum
  warnings.warn("Maximum Likelihood optimization failed to "
/home/sandbox/.local/lib/python3.11/site-packages/statsmodels/base/model.py:604: ConvergenceWarning: Maximum
  warnings.warn("Maximum Likelihood optimization failed to "
/home/sandbox/.local/lib/python3.11/site-packages/statsmodels/base/model.py:604: ConvergenceWarning: Maximum
  warnings.warn("Maximum Likelihood optimization failed to "
/home/sandbox/.local/lib/python3.11/site-packages/statsmodels/base/model.py:604: ConvergenceWarning: Maximum
  warnings.warn("Maximum Likelihood optimization failed to "
/home/sandbox/.local/lib/python3.11/site-packages/statsmodels/base/model.py:604: ConvergenceWarning: Maximum
  warnings.warn("Maximum Likelihood optimization failed to "
/home/sandbox/.local/lib/python3.11/site-packages/statsmodels/base/model.py:604: ConvergenceWarning: Maximum
  warnings.warn("Maximum Likelihood optimization failed to "
/home/sandbox/.local/lib/python3.11/site-packages/statsmodels/base/model.py:604: ConvergenceWarning: Maximum
  warnings.warn("Maximum Likelihood optimization failed to "
/home/sandbox/.local/lib/python3.11/site-packages/statsmodels/base/model.py:604: ConvergenceWarning: Maximum
  warnings.warn("Maximum Likelihood optimization failed to "
/home/sandbox/.local/lib/python3.11/site-packages/statsmodels/base/model.py:604: ConvergenceWarning: Maximum
  warnings.warn("Maximum Likelihood optimization failed to "
/home/sandbox/.local/lib/python3.11/site-packages/statsmodels/base/model.py:604: ConvergenceWarning: Maximum
  warnings.warn("Maximum Likelihood optimization failed to "
/home/sandbox/.local/lib/python3.11/site-packages/statsmodels/base/model.py:604: ConvergenceWarning: Maximum
  warnings.warn("Maximum Likelihood optimization failed to "
/home/sandbox/.local/lib/python3.11/site-packages/statsmodels/base/model.py:604: ConvergenceWarning: Maximum
  warnings.warn("Maximum Likelihood optimization failed to "
/home/sandbox/.local/lib/python3.11/site-packages/statsmodels/base/model.py:604: ConvergenceWarning: Maximum
  warnings.warn("Maximum Likelihood optimization failed to "
/home/sandbox/.local/lib/python3.11/site-packages/statsmodels/base/model.py:604: ConvergenceWarning: Maximum
  warnings.warn("Maximum Likelihood optimization failed to "
/home/sandbox/.local/lib/python3.11/site-packages/statsmodels/base/model.py:604: ConvergenceWarning: Maximum
  warnings.warn("Maximum Likelihood optimization failed to "
/home/sandbox/.local/lib/python3.11/site-packages/statsmodels/base/model.py:604: ConvergenceWarning: Maximum
  warnings.warn("Maximum Likelihood optimization failed to "
/home/sandbox/.local/lib/python3.11/site-packages/statsmodels/base/model.py:604: ConvergenceWarning: Maximum
  warnings.warn("Maximum Likelihood optimization failed to "
/home/sandbox/.local/lib/python3.11/site-packages/statsmodels/base/model.py:604: ConvergenceWarning: Maximum
```

```
  warnings.warn("Maximum Likelihood optimization failed to "
/home/sandbox/.local/lib/python3.11/site-packages/statsmodels/base/model.py:604: ConvergenceWarning: Maximun
  warnings.warn("Maximum Likelihood optimization failed to "
/home/sandbox/.local/lib/python3.11/site-packages/statsmodels/base/model.py:604: ConvergenceWarning: Maximun
  warnings.warn("Maximum Likelihood optimization failed to "
/home/sandbox/.local/lib/python3.11/site-packages/statsmodels/base/model.py:604: ConvergenceWarning: Maximun
  warnings.warn("Maximum Likelihood optimization failed to "
/home/sandbox/.local/lib/python3.11/site-packages/statsmodels/base/model.py:604: ConvergenceWarning: Maximun
  warnings.warn("Maximum Likelihood optimization failed to "
/home/sandbox/.local/lib/python3.11/site-packages/statsmodels/base/model.py:604: ConvergenceWarning: Maximun
  warnings.warn("Maximum Likelihood optimization failed to "
/home/sandbox/.local/lib/python3.11/site-packages/statsmodels/base/model.py:604: ConvergenceWarning: Maximun
  warnings.warn("Maximum Likelihood optimization failed to "
/home/sandbox/.local/lib/python3.11/site-packages/statsmodels/base/model.py:604: ConvergenceWarning: Maximun
  warnings.warn("Maximum Likelihood optimization failed to "
/home/sandbox/.local/lib/python3.11/site-packages/statsmodels/base/model.py:604: ConvergenceWarning: Maximun
  warnings.warn("Maximum Likelihood optimization failed to "
/home/sandbox/.local/lib/python3.11/site-packages/statsmodels/base/model.py:604: ConvergenceWarning: Maximun
  warnings.warn("Maximum Likelihood optimization failed to "
/home/sandbox/.local/lib/python3.11/site-packages/statsmodels/base/model.py:604: ConvergenceWarning: Maximun
  warnings.warn("Maximum Likelihood optimization failed to "
/home/sandbox/.local/lib/python3.11/site-packages/statsmodels/base/model.py:604: ConvergenceWarning: Maximun
  warnings.warn("Maximum Likelihood optimization failed to "
/home/sandbox/.local/lib/python3.11/site-packages/statsmodels/base/model.py:604: ConvergenceWarning: Maximun
  warnings.warn("Maximum Likelihood optimization failed to "
/home/sandbox/.local/lib/python3.11/site-packages/statsmodels/base/model.py:604: ConvergenceWarning: Maximun
  warnings.warn("Maximum Likelihood optimization failed to "
/home/sandbox/.local/lib/python3.11/site-packages/statsmodels/base/model.py:604: ConvergenceWarning: Maximun
  warnings.warn("Maximum Likelihood optimization failed to "
/home/sandbox/.local/lib/python3.11/site-packages/statsmodels/base/model.py:604: ConvergenceWarning: Maximun
  warnings.warn("Maximum Likelihood optimization failed to "
/home/sandbox/.local/lib/python3.11/site-packages/statsmodels/base/model.py:604: ConvergenceWarning: Maximun
  warnings.warn("Maximum Likelihood optimization failed to "
/home/sandbox/.local/lib/python3.11/site-packages/statsmodels/base/model.py:604: ConvergenceWarning: Maximun
  warnings.warn("Maximum Likelihood optimization failed to "
/home/sandbox/.local/lib/python3.11/site-packages/statsmodels/base/model.py:604: ConvergenceWarning: Maximun
  warnings.warn("Maximum Likelihood optimization failed to "
/home/sandbox/.local/lib/python3.11/site-packages/statsmodels/base/model.py:604: ConvergenceWarning: Maximun
  warnings.warn("Maximum Likelihood optimization failed to "
/home/sandbox/.local/lib/python3.11/site-packages/statsmodels/base/model.py:604: ConvergenceWarning: Maximun
  warnings.warn("Maximum Likelihood optimization failed to "
/home/sandbox/.local/lib/python3.11/site-packages/statsmodels/base/model.py:604: ConvergenceWarning: Maximun
  warnings.warn("Maximum Likelihood optimization failed to "
/home/sandbox/.local/lib/python3.11/site-packages/statsmodels/base/model.py:604: ConvergenceWarning: Maximun
  warnings.warn("Maximum Likelihood optimization failed to "
/home/sandbox/.local/lib/python3.11/site-packages/statsmodels/base/model.py:604: ConvergenceWarning: Maximun
  warnings.warn("Maximum Likelihood optimization failed to "
/home/sandbox/.local/lib/python3.11/site-packages/statsmodels/base/model.py:604: ConvergenceWarning: Maximun
  warnings.warn("Maximum Likelihood optimization failed to "
/home/sandbox/.local/lib/python3.11/site-packages/statsmodels/base/model.py:604: ConvergenceWarning: Maximun
  warnings.warn("Maximum Likelihood optimization failed to "
/home/sandbox/.local/lib/python3.11/site-packages/statsmodels/base/model.py:604: ConvergenceWarning: Maximun
  warnings.warn("Maximum Likelihood optimization failed to "
/home/sandbox/.local/lib/python3.11/site-packages/statsmodels/base/model.py:604: ConvergenceWarning: Maximun
  warnings.warn("Maximum Likelihood optimization failed to "
/home/sandbox/.local/lib/python3.11/site-packages/statsmodels/base/model.py:604: ConvergenceWarning: Maximun
  warnings.warn("Maximum Likelihood optimization failed to "
/home/sandbox/.local/lib/python3.11/site-packages/statsmodels/base/model.py:604: ConvergenceWarning: Maximun
  warnings.warn("Maximum Likelihood optimization failed to "
/home/sandbox/.local/lib/python3.11/site-packages/statsmodels/base/model.py:604: ConvergenceWarning: Maximun
  warnings.warn("Maximum Likelihood optimization failed to "
/home/sandbox/.local/lib/python3.11/site-packages/statsmodels/base/model.py:604: ConvergenceWarning: Maximun
  warnings.warn("Maximum Likelihood optimization failed to "
/home/sandbox/.local/lib/python3.11/site-packages/statsmodels/base/model.py:604: ConvergenceWarning: Maximun
  warnings.warn("Maximum Likelihood optimization failed to "
/home/sandbox/.local/lib/python3.11/site-packages/statsmodels/base/model.py:604: ConvergenceWarning: Maximun
  warnings.warn("Maximum Likelihood optimization failed to "
/home/sandbox/.local/lib/python3.11/site-packages/statsmodels/base/model.py:604: ConvergenceWarning: Maximun
  warnings.warn("Maximum Likelihood optimization failed to "
/home/sandbox/.local/lib/python3.11/site-packages/statsmodels/base/model.py:604: ConvergenceWarning: Maximun
  warnings.warn("Maximum Likelihood optimization failed to "
/home/sandbox/.local/lib/python3.11/site-packages/statsmodels/base/model.py:604: ConvergenceWarning: Maximun
  warnings.warn("Maximum Likelihood optimization failed to "
/home/sandbox/.local/lib/python3.11/site-packages/statsmodels/base/model.py:604: ConvergenceWarning: Maximun
  warnings.warn("Maximum Likelihood optimization failed to "
/home/sandbox/.local/lib/python3.11/site-packages/statsmodels/base/model.py:604: ConvergenceWarning: Maximun
  warnings.warn("Maximum Likelihood optimization failed to "
/home/sandbox/.local/lib/python3.11/site-packages/statsmodels/base/model.py:604: ConvergenceWarning: Maximun
  warnings.warn("Maximum Likelihood optimization failed to "
/home/sandbox/.local/lib/python3.11/site-packages/statsmodels/base/model.py:604: ConvergenceWarning: Maximun
```

```
  warnings.warn("Maximum Likelihood optimization failed to "
/home/sandbox/.local/lib/python3.11/site-packages/statsmodels/base/model.py:604: ConvergenceWarning: Maximum
  warnings.warn("Maximum Likelihood optimization failed to "
/home/sandbox/.local/lib/python3.11/site-packages/statsmodels/base/model.py:604: ConvergenceWarning: Maximum
  warnings.warn("Maximum Likelihood optimization failed to "
/home/sandbox/.local/lib/python3.11/site-packages/statsmodels/base/model.py:604: ConvergenceWarning: Maximum
  warnings.warn("Maximum Likelihood optimization failed to "
/home/sandbox/.local/lib/python3.11/site-packages/statsmodels/base/model.py:604: ConvergenceWarning: Maximum
  warnings.warn("Maximum Likelihood optimization failed to "
/home/sandbox/.local/lib/python3.11/site-packages/statsmodels/base/model.py:604: ConvergenceWarning: Maximum
  warnings.warn("Maximum Likelihood optimization failed to "
/home/sandbox/.local/lib/python3.11/site-packages/statsmodels/base/model.py:604: ConvergenceWarning: Maximum
  warnings.warn("Maximum Likelihood optimization failed to "
/home/sandbox/.local/lib/python3.11/site-packages/statsmodels/base/model.py:604: ConvergenceWarning: Maximum
  warnings.warn("Maximum Likelihood optimization failed to "
/home/sandbox/.local/lib/python3.11/site-packages/statsmodels/base/model.py:604: ConvergenceWarning: Maximum
  warnings.warn("Maximum Likelihood optimization failed to "
/home/sandbox/.local/lib/python3.11/site-packages/statsmodels/base/model.py:604: ConvergenceWarning: Maximum
  warnings.warn("Maximum Likelihood optimization failed to "
/home/sandbox/.local/lib/python3.11/site-packages/statsmodels/base/model.py:604: ConvergenceWarning: Maximum
  warnings.warn("Maximum Likelihood optimization failed to "
/home/sandbox/.local/lib/python3.11/site-packages/statsmodels/base/model.py:604: ConvergenceWarning: Maximum
  warnings.warn("Maximum Likelihood optimization failed to "
/home/sandbox/.local/lib/python3.11/site-packages/statsmodels/base/model.py:604: ConvergenceWarning: Maximum
  warnings.warn("Maximum Likelihood optimization failed to "
```

The table displays the empirical coverage probabilities of the 95% confidence interval for the log-odds ratio across ten different case-control sample size settings. The intervals are derived from logistic regression under the null hypothesis of no effect. Some warnings indicate occasional non-convergence in the logistic model due to small sample sizes, which is expected and acceptable in such simulations.
